# Rates of complete diagnostic testing for patients with acute myeloid leukemia

**DOI:** 10.1002/cam4.406

**Published:** 2015-01-26

**Authors:** Tara L Lin, Travis Williams, Jianghua He, Omar S Aljitawi, Siddhartha Ganguly, Sunil Abhyankar, Allan Fleming, Heather Male, Joseph P McGuirk

**Affiliations:** 1 Division of Hematology/Oncology, Department of Internal Medicine, University of Kansas School of Medicine3901 Rainbow Blvd., Kansas City, Kansas, 66210; 2 Department of Biostatistics, University of Kansas School of Medicine3901 Rainbow Blvd., Kansas City, Kansas, 66210

**Keywords:** AML, molecular testing, practice patterns

## Abstract

In addition to cytogenetics, additional molecular markers of prognosis have been identified and incorporated into the management of patients with acute myeloid leukemia (AML). We hypothesized that rates of molecular testing would be higher in an academic center versus community sites. A retrospective chart review included all de novo AML patients (excluding M3) at Kansas University Medical Center (KUMC) from January 2008 through April 2013. Records were evaluated for completeness of molecular testing as indicated by karyotype (FLT3, CEBPα, NPM1 in normal cytogenetics AML and c-KIT in core binding factor [CBF] AML). 271 charts were reviewed: 98 with CN-AML and 29 with CBF AML. Seventy were diagnosed at KUMC, 57 at a community site. Molecular testing was sent in 76/98 (77%) patients with CN-AML. Patients diagnosed at KUMC had a significantly higher rate of molecular testing (51/55, 93%) as compared to those diagnosed at outside centers (18/43, 41%) (*P* < 0.001). Of 29 patients with CBF AML, c-kit mutational analysis was performed more frequently at KUMC (14/15, 93%) than in community sites (8/14, 57%) (*P* = 0.035). There was a trend towards increased testing at both KUMC and community sites in later years. Rates of molecular testing in AML were higher in an academic center versus community sites in the 5 years following the World Health Organization revised classification of AML. All physicians who diagnose and treat AML must remain up to date on the latest recommendations and controversies in molecular testing in order to appropriately risk stratify patients and determine optimal therapy.

## Introduction

Acute Myeloid Leukemia (AML) is a heterogeneous disease with variable clinical outcomes to conventional chemotherapy. Long-term outcomes for patients treated with chemotherapy have not significantly improved in over thirty years, and the National Comprehensive Cancer Network (NCCN) recommends treatment on a clinical trial for newly diagnosed patients with AML when available 1. Although clinical features such as patient age, white blood cell count and history of prior chemotherapy or antecedent hematologic disorders predict patient prognosis, cytogenetic analysis at diagnosis remains the most important predictor of outcome. Approximately, 50% of patients with newly diagnosed AML will have a normal karyotype, classified as having an intermediate risk of relapse. In recent years, large clinical datasets identified molecular markers of prognosis particularly within this normal karyotype subset 2,3. The use of this additional molecular analysis has allowed for the further stratification of normal cytogenetics AML into groups with better and worse prognosis, guiding their clinical treatment. Mutations in fms-related tyrosine kinase 3 (FLT3) confer a worse prognosis, while mutations in nucleophosmin (NPM1) and CCAAT/enhancer-binding protein *α* (CEBP*α*) predict for prolonged remissions with chemotherapy alone. Patients with core binding factor [CBF] AML, inversion 16 or t(8;21), have traditionally been considered as “favorable risk” AML with disease relapse in “only” 40–50% of patients treated with chemotherapy alone. In 2006, the c-Kit mutation was described in patients with CBF and was predictive of increase in disease recurrence 4,5. The NCCN and European Leukemia Network (ELN) incorporate molecular testing specifically in normal cytogenetics and CBF leukemia karyotypes in prognostic categories and post-induction treatment recommendations 6. In 2008, the World Health Organization (WHO) revised its classification 7 of acute leukemia in order to recognize the impact that these molecular markers have on prognosis in patients with normal cytogenetics and recommend their widespread use. Because the majority of patients with AML will achieve complete remission with induction chemotherapy, collection and complete diagnostic and prognostic testing of the bone marrow at time of diagnosis is critical to predicting outcomes and determining post-induction treatment strategies. Thus, we studied records from patients with AML from 2008 (the year of the revised WHO classifications) seen at our center and measured rates of completeness of diagnostic testing in patients diagnosed at our academic center as compared to those diagnosed in the community.

## Methods

A retrospective chart review of all newly diagnosed AML patients (excluding subtype M3 or acute promyelocytic leukemia) that were seen at Kansas University Medical Center (KUMC) from 1 January 2008 through 1 April 2013 was performed. This research was approved by the Human Subjects Committee at the University of Kansas Medical Center. Records were evaluated for completeness of acquiring diagnostic information including age, gender, cytogenetics and molecular testing, if indicated by karyotype (FLT3, CEBP*α*, and NPM1 in normal cytogenetics and c-KIT in CBF leukemia). These data were collected for patients diagnosed at KUMC or referred to KUMC by community practices either for initial chemotherapy or evaluation for stem cell transplant. Statistical significance for the comparisons between KUMC and community sites was obtained using the chi-square test or the Fisher's exact test when patient numbers were small.

## Results

A total of 271 charts were reviewed. In all but one case, there was complete conventional cytogenetic information available from the diagnostic bone marrow aspiration (in one case, there was not sufficient chromosome growth for cytogenetic analysis). Among a total of 127 charts analyzed for completeness of molecular testing, 98 patients had normal cytogenetics and 29 patients had CBF leukemia; 70 patients were diagnosed at KUMC and 57 were diagnosed at a community site. Molecular testing was sent for 76/98 (77%) patients with normal cytogenetics at all sites (Table1). Patients diagnosed at KUMC had a significantly higher rate of molecular testing (51/55, 93%) as compared to those diagnosed at referring centers (18/43, 41%, *P* < 0.001). Of 29 patients diagnosed with CBF leukemia, c-kit mutational analysis testing was done more frequently at KUMC (14/15, 93%) than in community sites (8/14, 57%, *P* = 0.035). When we examined the rates of molecular testing by year, there was an increase in testing over time at both KUMC and community sites; however, the numbers per year in each setting were too small to analyze for statistical significance. In the 5 year time period from the WHO revision of AML classification in 2008, rates of molecular testing for patients with CBF and normal cytogenetics AML were higher at our academic center versus community referring sites, although rates of molecular testing in the community increased over that time frame.

**Table 1:** Rates of molecular testing in AML

	2008	2009	2010	2011	2012
Core Binding Factor AML c-KIT mutation testing (# samples tested/total CBF AML per year)					
Academic	0/1	1/1	3/3	6/6	4/4
Community	0/1	3/4	3/6	0/0	2/3
Normal cytogenetics AML FLT3, NPM1, CEBPA testing (# samples tested/total normal cytogenetics AML per year)					
Academic	2/4	8/10	12/12	11/11	18/18
Community	0/8	5/7	7/13	9/9	4/6

## Discussion

Few studies have specifically examined the differences in outcomes of patients treated at an academic center versus community-based practice 8 Limited studies have shown improved surgical outcomes in patients treated at an (National Cancer Institute) NCI-designated cancer center compared to hospitals without NCI-designation 9–12, but the data are scarce in the medical specialties. In hematologic malignances, there are few reports comparing evaluation, treatments, and outcomes of patients treated in the community versus academic centers. One British population-based study of childhood leukemia showed no difference in overall survival for patients treated at a teaching hospital or in the community, and no advantage for patients treated on clinical trials 13. A review of centers performing stem cell transplantation found significantly decreased overall survival at centers performing fewer than 5 matched sibling donor transplants per year 14. More recently, preliminary data on a registry of patients with myelodysplastic syndrome in Minnesota found that patients treated at an academic center received more aggressive therapy than those treated in the community 15. Our data are the only report which describes that rates of complete diagnostic evaluation, including molecular testing, for newly diagnosed patients with AML is higher in an academic center versus community practices.

Although this chart review is limited in that we were unable to review charts of AML patients who were diagnosed and received their entire treatment in the community or were referred to other academic centers, it does provide information regarding rates of adoption of guidelines for molecular testing in AML. As molecular testing is becoming more commercially available and its significance in predicting AML outcomes more well-known, the rates of molecular testing in all settings are expected to increase. A major concern continues to be the cost benefit ratio, as additional molecular markers are being described but with unclear therapeutic interventions to address the increased risk of relapse in some subtypes. There are clinical trials of targeted therapies for patients with mutations in FLT3 and c-KIT, with variable clinical benefit. Large datasets have found mutations in additional genes (including IDH1/2, DNMT3A, RUNX1, TET2, NRAS, MLL, and others) to confer adverse prognosis, but it remains unclear how modifications to treatment regimens or the use of stem cell transplantation will impact outcomes in these specific subtypes of AML. It is challenging to keep up with this growing body of literature and how to incorporate these new markers into routine clinical practice remains controversial. It is critically important that all physicians who diagnose and treat AML remain current on the latest recommendations in molecular testing in order to appropriately risk stratify patients and recommend appropriate postremission therapies for patients. The emerging data on these molecular markers of prognosis underscores the importance of enrolling patients with AML on clinical trials whenever possible. Our data suggest that patients treated at an academic center are more likely to have complete molecular testing performed at diagnosis, and, therefore, better informed clinical decision making regarding choice of postremission therapy.
